# Optical Logic Gates Excited by a Gauss Vortex Interference Beam Based on Spatial Self-Phase Modulation in 2D MoS_2_

**DOI:** 10.3390/nano13081423

**Published:** 2023-04-20

**Authors:** Xueyu Chen, Ge Ding, Linwei Tang, Haijian Zou, Chaofeng Wang, Shuqing Chen, Chenliang Su, Ying Li

**Affiliations:** 1 International Collaborative Laboratory of 2D Materials for Optoelectronics Science & Technology of Ministry of Education, Engineering Technology Research Center for 2D Material Information Function Devices and Systems of Guangdong Province, Institute of Microscale Optoelectronics, Shenzhen University, Shenzhen 518060, China; 2 BYD Semiconductor Company Limited, Shenzhen 518060, China

**Keywords:** optical logic gate, spatial self-phase modulation, interference light, diffraction rings, multidimensional modulation, optical logic operation

## Abstract

Vortex beams with optical orbital angular momentum have broad prospects in future high-speed and large-capacity optical communication. In this investigation of materials science, we found that low-dimensional materials have feasibility and reliability in the development of optical logic gates in all-optical signal processing and computing technology. We found that spatial self-phase modulation patterns through the MoS_2_ dispersions can be modulated by the initial intensity, phase, and topological charge of a Gauss vortex superposition interference beam. We utilized these three degrees of freedom as the input signals of the optical logic gate, and the intensity of a selected checkpoint on spatial self-phase modulation patterns as the output signal. By setting appropriate thresholds as logic codes 0 and 1, two sets of novel optical logic gates, including AND, OR, and NOT gates, were implemented. These optical logic gates are expected to have great potential in optical logic operations, all-optical networks, and all-optical signal processing.

## 1. Introduction

The increasing demand of various Internet-based applications for higher bandwidth and ultra-fast operation speed is currently facing an electronics bottleneck problem. Compared with electrons, light has several advantages, such as higher speed, greater capacity, and fewer interactions. Although optical fiber cables are used in communication systems, the conversion of light to electricity reduces operational speed and, therefore, cannot fully utilize the capacity of optical systems [1,2]. Therefore, all-optical signal processing, all-optical devices, and complete optical networks have been identified as potential candidates to replace conventional electronic-integrated circuits.

To form a complete optical network, all operations, from signal generation, processing, encoding, modulation, transmission, demodulation, decoding, filtering, etc., must be performed in an all-optical manner. The various devices used to perform these operations are electronic-based and must be switched to a complete optical domain. This requires all-optical logic gates, all-optical timing and combination circuits, all-optical processors, etc. In developing such optical circuits, the design of all-optical logic gates is a prerequisite. The design of such gates needs to consider various design aspects [3]. For example, the optical logic gate based on polarization coding selects the horizontal linear polarization state and the vertical linear polarization state of the optical signal to represent logic 1 and logic 0, respectively. By selecting the polarization state of the incident light and the applied voltage as two binary logic signals and transmitting the orthogonal polarization state, an optical logic gate based on the periodically polarized lithium niobate (PPLN) electro-optic effect was finally achieved [4,5,6]. Existing optical logic gates typically have limitations in their degree of freedom of adjustment; accordingly, they may not be able to meet the growing capacity requirements. Therefore, how to improve the degree of freedom of optical logic gates is one of the key issues in expanding the application of optical logic gates.

The optical response of two-dimensional (2D) nanomaterials has attracted much attention due to their powerful nonlinear properties and potential applications of broadband Kerr nonlinearity in photonic devices [7,8]. When the strong laser passes through a 2D material suspension, the strong coherent light–matter interaction creates spatial phase modulation of the incident laser and produces self-diffraction ring patterns in the far field. This phenomenon, called spatial self-phase modulation (SPPM), can be used to measure nonlinear optical parameters and achieve optical switching [9,10,11,12].

The currently reported optical switcher based on the spatial self-phase modulation (SSPM) effect of 2D materials is realized by changing the number of diffraction rings at different powers, and it can only achieve single-dimensional power modulation [13,14,15,16]. This limitation hinders the further application of 2D materials’ nonlinear optical switches. Because the spatial nonlinear modulation of 2D materials is related to the distribution of spatial light intensity, it is expected to provide more dimensional modulation by introducing beams with a non-uniform spatial light intensity distribution. A vortex beam is a spatial structure beam with annular intensity distribution, phase singularity and orbital angular momentum (OAM). Vortex beams have been widely used in particle manipulation and optical communication [17,18,19]. Laguerre–Gaussian mode vortex beams are characterized by spiral phase factor $\exp\left(il\theta \right)$, where θ is the azimuth angle and l is an integer number called the topological charge (TC), referring to OAM equivalent to lℏ carried per photon. Furthermore, the interference patterns of vortex beams and Gaussian beams are in a petaloid structure, which may provide a possibility for exciting new SSPM to realize multi-dimensional optical switches and logic gates.

In this work, we propose a set of basic optical logic gates based on the dispersion SSPM of an MoS_2_ nano-panel. By modulating the incident power, initial phase, and the TC of the incident beam, we can realize the radial modulation and angular modulation of the diffraction rings, respectively. Based on this, the basic logic AND gate, OR gate, and NOT gate are realized. In this study, when the Gauss vortex superposed interference light with a spiral shape passes through the nonlinear material and excites the SSPM effect, the spiral beam expands and deforms to form the special diffraction rings. It can be easily found that the interference pattern rotates with the variety of the initial phase of the vortex beam, so it can be used to design an optical switcher with angular modulation. Similar to common SSPM-based switches, the on–off regulation can also be realized by increasing the power of incident light when the diffraction rings do not rotate. It can also be found that the number of spiral beam tails is consistent with the TC of the vortex beam, so we can adjust the spiral-shaped pattern by changing the TC. The results show that the interference beam formed by the interference of the Gaussian beam and the vortex beam can realize multi-dimensional an optical switcher and optical logic gates through a nonlinear diffraction device composed of MoS_2_ nanosheets dispersion, and has great potential in optical computing and optical transmission applications.

## 2. Preparation and Nonlinear Property of MoS_2_

### 2.1. Preparation and Characterization

Few-layer MoS_2_ dispersions were prepared by the lithium intercalation exfoliation method [20,21]. Lithium intercalation was achieved by immersing 3 g of natural MoS_2_ crystals in 3 mL of 1.6 M hexane butyllithium solution acquired from the Sigma-Aldrich Corporation, then soaking in a bottle filled with argon gas for 2 days. The Li_x_MoS_2_ was recovered by filtration and washed with hexane (60 mL) to remove excess lithium and organic residues. After that, the Li_x_MoS_2_ was used to perform ultrasonication in water for 1 h to obtain peeling (to avoid disengagement within 30 min). The mixture was centrifuged several times to remove excess lithium in the form of LiOH and un-exfoliated material. After removing the thicker MoS_2_ blocks, the few-layer 1T-2H hybrid MoS_2_ dispersions were obtained and used for the following experiments.

**Figure 1:**
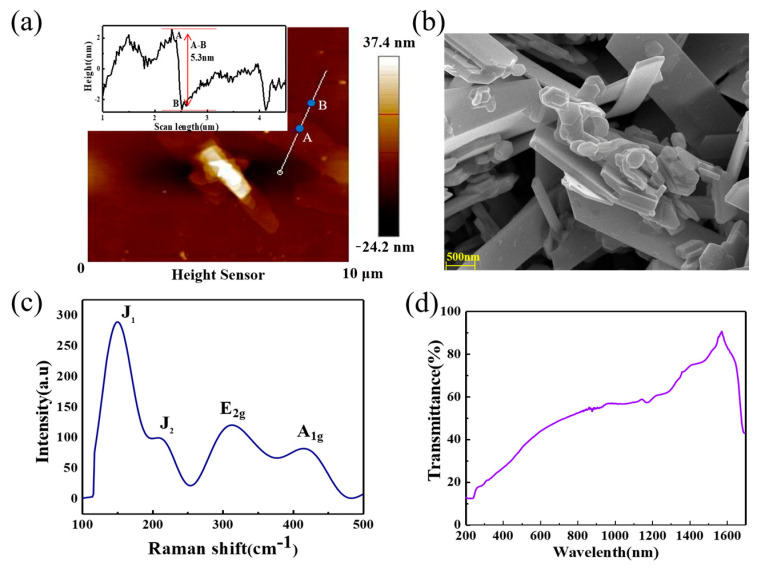
Basic characterizations of MoS_2_ nanosheets: (**a**) test result of atomic force microscope (AFM); inset: height curve of the white line; (**b**) scanning electron microscope (SEM) image of MoS_2_ nanosheets; (**c**) Raman spectrum of MoS_2_ nanosheets; (**d**) transmittance spectrum of the MoS_2_ nanosheets.

An atomic force microscope (AFM) and a scanning electron microscope (SEM) were used to characterize the morphology of 1T-2H hybrid MoS_2_ nanosheets. An AFM image was used to depict the topographic morphology. As shown in Figure 1a, the thickness of MoS_2_ nanosheets were nearly 5.3 nm, which is about 6 layers because the thickness of monolayer MoS_2_ is estimated to be 0.85 nm. In the SEM image in Figure 1b, the lateral size of the MoS_2_ nanosheet is shown. Raman spectroscopy is commonly used to analyze and identify materials. Here, the samples were placed on a Si wafer after diluting the initial dispersions; the Raman spectra of the MoS_2_ nanosheets in the region of 100–500 cm^−1^ are shown in Figure 1c. The two phonon modes of E_2g_ and A_1g_ appeared at 311.7 cm^−1^ and 416.9 cm^−1^, which indicated that the prepared MoS_2_ nanosheet was less than 10 layers. Moreover, a characteristic mode of 1T MoS_2_ was also observed as additional peak J_1_ at 150.4 cm^−1^ and J_2_ at 208.1 cm^−1^, which can be explained as some MoS_2_ nanosheets are formed as $2a_{0}\times a_{0}$ superlattice of octahedral in lithiation. The Brillouin zone of nanosheets folds in superlattice and Γ point coincides boundary. Therefore, the J_1_ and J_2_ peaks are corresponding to phonons at M points of the Brillouin zone [22,23]. Figure 1d shows that the MoS_2_ nanosheets had a broadband absorption ranging from 200 nm to 1600 nm, which may have potential in designing ultraviolet visible infrared optical devices.

### 2.2. Nonlinear Property of MoS_2_

**Figure 2:**
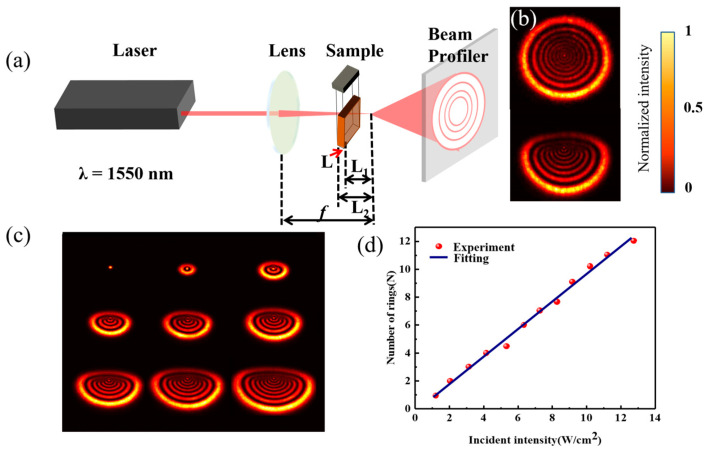
Experimental analysis of nonlinearity of few-layer MoS_2_: (**a**) schematic diagram of the experimental setup; (**b**) diffraction rings pattern at maximum and distorted condition; (**c**) the variation of diffraction rings with increasing intensity; (**d**) the number of diffraction rings linearly related with incident intensity.

The change in refractive index caused by light modulates the phase of the incident light, increases the nonlinear phase shift of the beam on the cross-section, and finally forms a diffraction ring between the bright and dark phases in the far-field distribution. A nonlinear diffraction device is shown in Figure 2a. High-energy light was emitted from the laser (λ = 1550 nm), focused by a lens (f = 200 mm), and then directed into the MoS_2_ dispersion. The SSPM was excited to generate a diffraction ring and the spot pattern was finally collected by a laser beam profiler.

After passing through MoS_2_ nanosheets, a diffraction ring gradually developed on the receiving screen, as shown in Figure 2b. As the diffraction ring reached its maximum, the upper half of the rings collapsed toward the center. The vertical direction was more distorted than the horizontal direction. In other words, the diffraction rings remained stable in the lower half, but collapsed in the upper half. According to Wang et al. [24], this collapse (in the upper half) occurs due to non-axis-symmetrical thermal convection. Dispersive media absorb part of the propagating laser light because of their finite optical absorption coefficients [25,26]. Consequently, thermal convection is enhanced around the laser spot as the temperature gradient increases along vertical direction [27]. Due to the above convection process, there is less concentration of MoS_2_ nanosheets in the upper part of the medium than in the lower part. Hence, the dispersed MoS_2_ nanosheets distort the diffraction patterns at the upper part of the laser beam, causing them to collapse toward the center.

SSPM, a third-order nonlinear effect that induces the Kerr effect, derives from the nonlinear phase shift generated by the photoelectric field in the medium through third-order nonlinear susceptibility. It usually has been employed to measure the optical nonlinear properties of materials. For devices based on nonlinear effects, the value of the nonlinear refractive index coefficient directly affects the performance of the device, so it is necessary to calculate the nonlinear refractive index of MoS_2_. According to the nonlinear Kerr effect, the refractive index of few-layer MoS_2_ can be defined as:

(1) $$n=n_{0}+n_{2}I$$

where $n_{0}=1.486$ is the linear refractive index, which is approximately equal to the refractive index of NMP, $n_{2}$ is the nonlinear refractive index of the MoS_2_, and I is the intensity of the incident light [28].

When a strong beam passes through a small layer of MoS_2_ dispersion, SSPM occurs, and its nonlinear phase shift can be defined as [29,30]:

(2) $$\Delta \phi =\frac{2\pi n_{0}}{\lambda }\int_{0}^{L_{eff}}n_{2}I(r,z)dz$$

Here, $L_{eff}$ represents the effective optical thickness of the few-layer MoS_2_ nanosheets, r represents the radial coordinates, and I(r,z) represents the intensity distribution. $L_{eff}$ could be expressed as follows [31]:

(3) $$L_{eff}={\left.\;z_{0}\arctan(\frac{z}{z_{0}})\right|}_{L_{1}}^{L_{2}},(z_{0}=\frac{\pi \omega_{0}^{2}}{\lambda })$$

$L_{1}$ and $L_{2}$ represent, respectively, the distance from the focal point to sides of the quartz cuvette. The $L=L_{2}-L_{1}$ represents the thickness of the quartz cuvette. In addition, for a Gaussian beam, the center intensity is I(0,z)=2I (I is the average intensity). The relation satisfied by diffraction rings is [Δϕ(0)−Δϕ(∞)]=2Nπ. According to the above relations, the nonlinear refractive index $n_{2}$ can be expressed as [32]:

(4) $$n_{2}=\frac{\lambda }{2n_{0}L_{eff}}\cdot \frac{N}{I}$$

The third-order nonlinear susceptibility $\chi_{total}^{\left(3\right)}$ is defined as:

(5) $$\chi_{total}^{\left(3\right)}=\frac{c\lambda n_{0}}{2.4\times 10^{4}\pi^{2}L_{eff}}\cdot \frac{dN}{dI}$$

As shown in Figure 2c, as the power of the light source is increased, the number of diffraction rings increases. As depicted in Figure 2d, the number of diffraction rings and the light intensity have a linear relationship with the slope of $dN/dI=0.978{\;\mathrm{cm}}^{2}W^{-1}$. According to the above formula, the nonlinear refractive index and the third-order nonlinear susceptibility of the few-layer MoS_2_ nanosheets dispersion at 1550 nm are calculated, respectively, to be $n_{2}=5.33\times 10^{-5}{\;\mathrm{cm}}^{2}W^{-1}$. These results and values allowed us to analyze the nonlinear optical response of MoS_2_ and were very helpful in our all-optical logic gates study based on SSPM.

## 3. Gauss Vortex Interference and Nonlinear Modulation

### 3.1. The Physical Model of Gauss Vortex Interference

If a Gaussian beam with a wavelength of λ propagates along the z direction, then the light field when transmitted to a certain location can be expressed as:

(6) $$E(r,z)=A_{0}\frac{w_{0}}{w(z)}\exp(-\frac{r^{2}}{w{\left(z\right)}^{2}})\exp[-ikz-ik\frac{r^{2}}{2R(z)}+i\tau (z)]$$

where $w_{0}$ is the beam waist and the Rayleigh distance is $z_{0}=\pi w_{0}/\lambda$.

(7) $$w(z)=w_{0}{\left[1+{\left(z/z_{0}\right)}^{2}\right]}^{1/2}$$

(8) $$R(z)=z[1+{\left(z_{0}/z\right)}^{2}]$$

(9) $$\tau (z)=\tan^{-1}(z/z_{0})$$

where $A_{0}$ is a constant, k=2π/λ is the absolute value wave vector, w(z) is the beam width, and R(z) and τ(z) are the curvature radius of wavefront and phase delay, respectively.

As a magical beam, the vortex beam rotates in a spiral and has a dark core at the center with zero intensity and singularity. The vortex beam also has OAM, which is its most important feature. This feature is related to the TC, so the vortex beam propagating along the *z*-axis can be simplified to the cylindrical coordinate system according to different TCs [33]:

(10) $$E(r,\theta ,z)=E_{0}(r,\theta ,z)\exp(-il\theta )\exp(-ikz)$$

where $E_{0}$ represents the amplitude intensity, l indicates the number of TCs, which can be an integer or a fraction, and θ indicates the azimuth angle. In this experiment, a Gaussian beam was generally used to generate a vortex beam through a vortex phase plate, so the mathematical expression of the vortex beam field is [34,35]:

(11) $$E(r,\theta ,z)=A_{0}\frac{w_{0}}{w(z)}\exp(-\frac{r^{2}}{w{\left(z\right)}^{2}})\exp[-ikz-ik\frac{r^{2}}{2R(z)}+i\tau (z)]\exp(il\theta )$$

when the Gaussian beam interferes with the vortex beam, the expression can be simplified to:

(12) E=aE(r,z)+bE(r,θ,z)

Of course, the interference of the two beams is realized in the cylindrical coordinate system, and the intensity of the interference can be expressed as:

(13) $$I={\left|E\right|}^{2}={\left|aE(r,z)+bE(r,\theta ,z)\right|}^{2}$$

where the relationship between a and b is 0<a+b=1, indicating the proportions of the Gaussian beam and the vortex beam.

**Figure 3:**
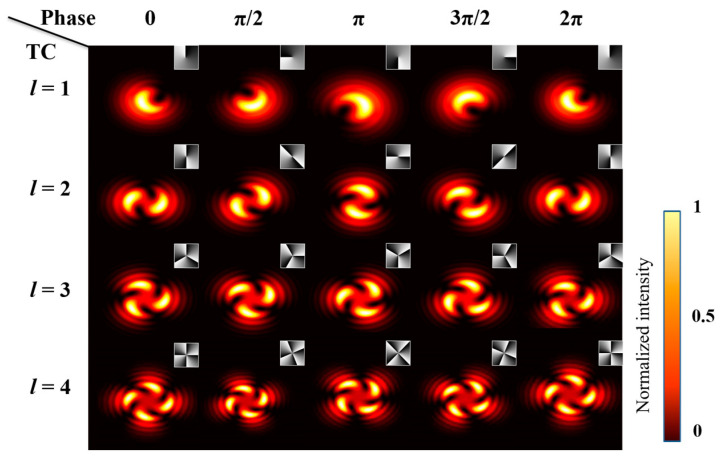
The simulated far-field intensity distribution of the Gaussian vortex interference light after nonlinear phase shift modulation. Inset: the phase of the spatial light modulation (SLM) load.

Figure 3 shows the interference intensity pattern of a Gaussian beam and a vortex beam. The specific parameter is a=b=0.5; the two beams are interfered and then transmitted coaxially for 30 cm for reception. The figure shows, from the simulation pattern, that the far-field distribution extends spiral tails around the periphery of the Gaussian light, and the number of tails is determined by the TC. The position of a tail is determined by the initial phase of the vortex beam.

### 3.2. Spatial Nonlinear Modulation Model of Gauss Vortex Interference

From Equation (6), the mathematical expression of the Gaussian beam transmitted to the *z*-axis after converging through a lens is:

(14) $$E(r,z)=A_{0}\frac{w_{0}}{w(z)}\exp(-\frac{r^{2}}{w{\left(z\right)}^{2}})\exp[-ikz-ik\frac{r^{2}}{2R(z)}+i\tau (z)]\exp(-ik\frac{r^{2}}{2f})$$

where f is the focal length of the converging lens. When we define the direction of propagation of the beam as the *z*-axis and the beam waist as the origin of the coordinate, then the lateral phase shift produced by the Gaussian beam passing through a nonlinear medium of thickness L is:

(15) $$△\phi (r)=k\int_{z^{\prime }}^{z^{\prime }+L}△n\left(r,z\right)dz=\;k\int_{z^{\prime }}^{z^{\prime }+L}n_{2}I\left(r,z\right)dz$$

$I\left(r,z\right)$ is the light intensity of SSPM excitation:

(16) $$I\left(r,z\right)={\left|E\left(r,z\right)\right|}^{2}$$

According to the Fraunhofer approximation based on the Fresnel–Kirchhoff diffraction formula, the electric field distribution that the Gaussian beam excites in the SSPM effect is:

(17) $$E\left(r,△z\right)=FFT^{-1}\{H\left(f_{x},f_{y}\right)\cdot FFT[E\left(r,z\right)\times \exp\left(i△\phi \left(r\right)\right)]\}$$

(18) $$H\left(f_{x},f_{y}\right)=\exp\left(-j\pi \lambda △z\left(f_{x}^{2}+f_{y}^{2}\right)\right)$$

Here, H is the transfer function based on Fresnel–Kirchhoff theory and $FFT^{-1}$ represents two-dimensional inverse Fourier transform.

Based on Equation (6), the electric field distribution of vortex beam focused by a lens is:

(19) $$\begin{array}{cc} E(r,\theta ,z)= & A_{0}\frac{w_{0}}{w(z)}\exp(-\frac{r^{2}}{w{\left(z\right)}^{2}})\exp[-ikz-ik\frac{r^{2}}{2R(z)}+i\tau (z)]\\ & \times \exp\left(-il\theta \right)\exp(-ik\frac{r^{2}}{2f}) \end{array}$$

The lateral additional phase shift is generated by the vortex beam converging on and passing through a nonlinear medium of thickness L:

(20) $$△\phi (r,\theta )=k\int_{z^{\prime }}^{z^{\prime }+L}△n\left(r,\theta ,z\right)dz=\;k\int_{z^{\prime }}^{z^{\prime }+L}n_{2}I^{\prime }\left(r,\theta ,z\right)dz$$

$I^{\prime }\left(r,\theta ,z\right)$ is the light intensity of the SSPM excitation:

(21) $$I^{\prime }\left(r,\theta ,z\right)={\left|E^{\prime }\left(r,\theta ,z\right)\right|}^{2}$$

The electric field distribution after Fresnel–Kirchhoff diffraction is:

(22) $$E\left(r,\theta ,△z\right)=FFT^{-1}\{H\left(f_{x},f_{y}\right)\cdot FFT[E\left(r,\theta ,z\right)\times \exp\left(i△\phi \left(r,\theta \right)\right)]\}$$

(23) $$H\left(f_{x},f_{y}\right)=\exp\left(-j\pi \lambda △z\left(f_{x}^{2}+f_{y}^{2}\right)\right)$$

When the Gauss vortex superposed interference light passes through the nonlinear material and excites the SSPM effect, the electric field expression of superposed interference light is:

(24) $$E^{\prime }(r,\theta ,△z)=E\left(r,△z\right)+E\left(r,\theta ,△z\right)$$

Finally, the far-field intensity distribution of the interference light is:

(25) $$I\left(r,\theta ,△z\right)={\left|\left(r,△z\right)+E\left(r,\theta ,△z\right)\right|}^{2}$$

where △z is the distance from the internal focus of the sample to the receiving screen.

**Figure 4:**
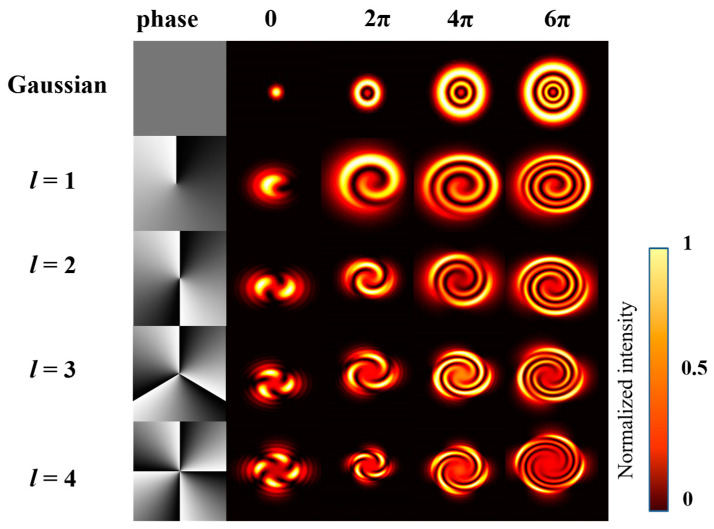
The simulated far-field intensity distribution of Gaussian beam and interference beam after nonlinear phase-shift modulation. The first column are the phase patterns loaded on the spatial phase modulator.

Combined with the above formulae, the SSPM effect can be generated and described. Figure 4 shows the simulated intensity distribution of diffraction rings when a normal Gaussian beam and a Gauss vortex interference beam with different phase shift modulation. For ordinary Gaussian light (see the first line of Figure 4), the figure shows from the simulation that the number of diffraction rings have a proportional relationship with the phase-shift modulation; each additional 2π phase shift will increase one more diffraction ring. On lines 2–5 of Figure 4, when a different lateral phase shift is added to the interference beam, the diffraction rings are also successfully excited and changed in the same manner as the Gaussian beam. The spiral beam expands with adding phase shifts, but the number of tails is the same as the value of the TC. In addition, note that the result of the interference beam with a negative TC is also the same, except that the direction of the tails becomes reversed.

## 4. Experiment Results and Discussion

### 4.1. The Interference Beam Generation and SSPM

**Figure 5:**
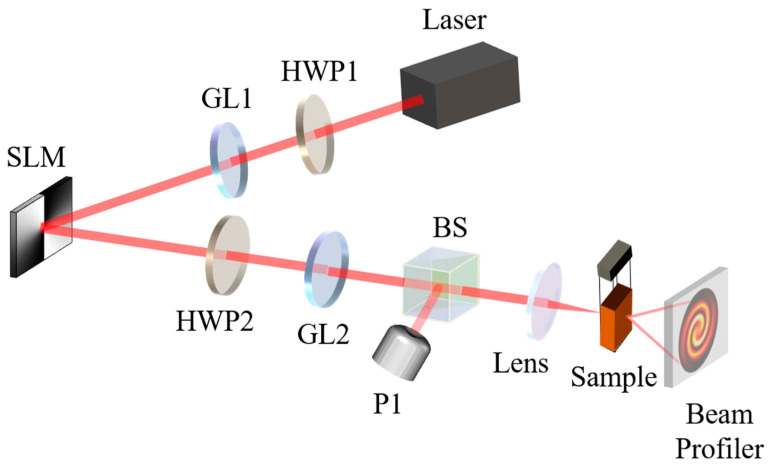
Experimental system for generating interference light and SSPM (Laser: CW laser; GL: Glan polarizer; SLM: spatial light modulator; HWP: half-wave plate; BS: beam splitter; P1: Power meter detector; Lens: focusing lens; Sample: MoS_2_ dispersion; beam profiler: charge coupled device beam analyzer).

Figure 5 shows the experimental system for generating an interference beam and the SSPM effect. The high intensity light with a wavelength of 1550 nm was emitted from the light source and passed through a half-wave plate (HWP1) and a Glan polarizer (GL1) to obtain the maximum light intensity (polarization direction at 45° to the *x*-axis). Then, it entered the SLM, which was loaded with a phase pattern to convert the Gaussian beam into a vortex beam. It should be noted that the SLM only works on horizontally polarized light. Due to the fast axis of GL1, set to 45°, the polarization of the incident beam can be regarded as the superposition of horizontal and vertical polarization. Hence, only the horizontal component was converted to vortex beams by SLM, while the vertical component remained unchanged as the Gaussian beam. Then, the polarized direction of the mixed beam was transformed back to 45° by a second half-wave plate (HWP2) with a 22.5° fast axis. At that point, an interference light of the Gaussian beam and the vortex light was obtained. Another Glan polarizer (GL2) was placed behind the HWP2 as a polarized filter and light intensity modulator to control the incident intensity of MoS_2_ dispersion by tuning the angle difference between its fast axis and the polarized direction of the interference beam. A beam splitter (BS) with 50:50 split ratio is placed behind GL2 to learn the incident power of Sample by detecting the power of equationally divided beam with a power meter detector (P1). The interference light was focused on the MoS_2_ dispersion (sample) through a lens (f = 200 mm), and excited by the interaction between light and matter to form a spiral diffraction pattern, which was finally received by a laser beam profiler.

**Figure 6:**
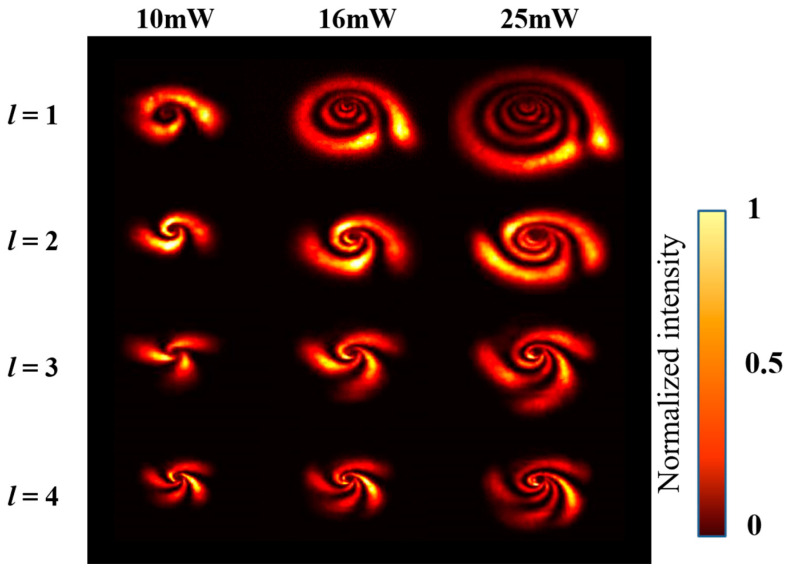
The experimental far-field distribution of interference beam based on SSPM. The vertical axis represents the interference beam with different TCs. The horizontal axis represents different power.

As shown in Figure 5 in the dashed area, when the interference beam passes through the MoS_2_, the SSPM effect can be excited and the interference beam pattern expands and distorts; the results are shown in Figure 6. The far-field distribution at powers of 10 mW, 16 mW, and 25 mW is shown in the horizontal direction and the far-field distribution of different TCs is shown in the longitudinal direction. It can be clearly seen from the data that the number of diffraction rings of the spiral beam is consistent with the nonlinear phase excitation by the strong beam intensity, and the number of tails is consistent with the TC. Incidentally, the interaction between light and MoS_2_ dispersion produces the collapse effect of the diffraction rings, then produces deformation induced by the gravity and the thermal effects. These results indicate that the spiral beam generation and the SSPM effect have been realized experimentally, and the experimental results are consistent with the theoretical results.

**Figure 7:**
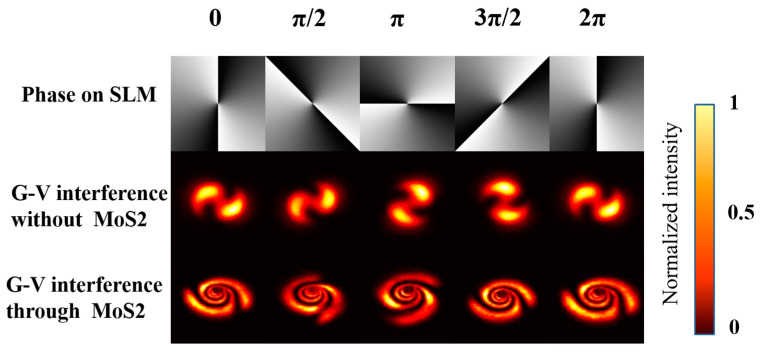
The experimental far field of Gauss vortex interference beam with phase change at a fixed power (25 mW) and a fixed TC (*l* = 2).

Figure 7 shows the far-field distribution of a Gauss vortex interference beam at a fixed power (25 mW) when the interference light has different initial phases. If the topological charge of the interference light is set to 2 and the initial phase is modulated from 0 to 2π, the modulation interval is 1/2π. It can be observed that changing the initial phase causes changes in the diffraction pattern, independent of the SSPM effect and consistent with previous simulation results.

### 4.2. Multi-Dimensional Optical Switcher

**Figure 8:**
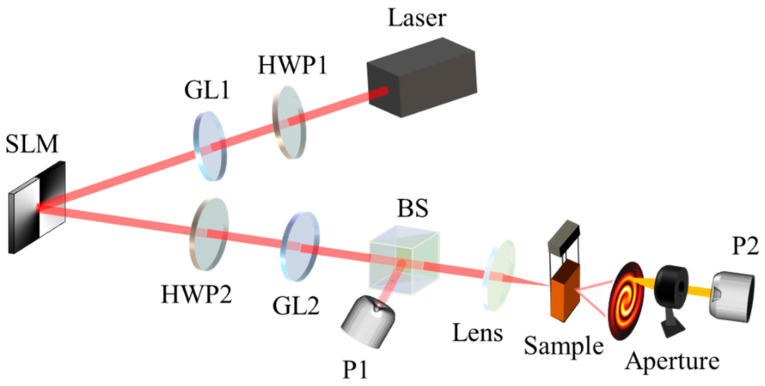
Schematic diagram of multi-dimensional optical switcher system (Laser: CW laser; HWP1 and HWP2: half-wave plate; GL1 and GL2: Glan polarizer; SLM: spatial light modulator; BS: beam splitter; P1 and P2: Power meter detector; Lens: focusing lens; Sample: MoS_2_ dispersion; Aperture: beam aperture).

According to the previous description, we found that the diffraction rings changed with the incident light intensity and the initial phase, so we produced the optical switcher based on exciting the SSPM effect and modulating the light intensity and the initial phase. The experimental device is shown in Figure 8. The interference beam was split into two beams by a 50:50 beam splitter. One beam was received by P1 to record real-time power; the other beam was subjected to SSPM, and an aperture was placed at the rear end of the diffraction rings. P2 was placed behind the aperture for receiving power.

**Figure 9:**
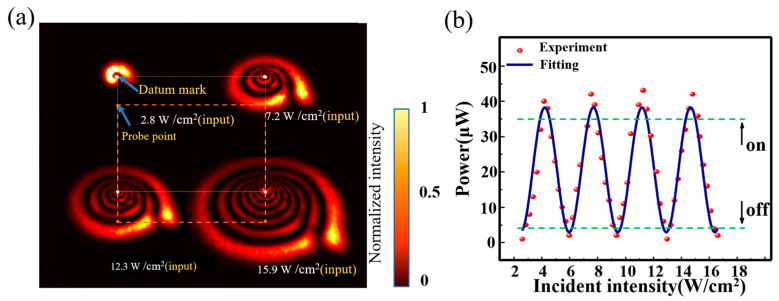
Operation principles of power-triggered optical switching: (**a**) diffraction rings at different powers (Orange dotted lines: indicating the relative position of the probe point and datum mark); (**b**) power variation at the probe point with increasing input signal power (Green dotted lines: threshold of “on” and “off” level signal).

As shown in Figure 9a, as the power of the light source was increased, the number of diffraction rings of the spiral beam increased. When an aperture was placed at the orange dots position, as shown in Figure 9a, the power gradually increased, and the transmission power of the aperture exhibited a continuous change in peaks and valleys. The data results are shown in Figure 9b. If a certain threshold was set as the critical point of the switcher, then the purpose of a continuous optical switcher could be achieved by modulating the power of the incident light.

**Figure 10:**
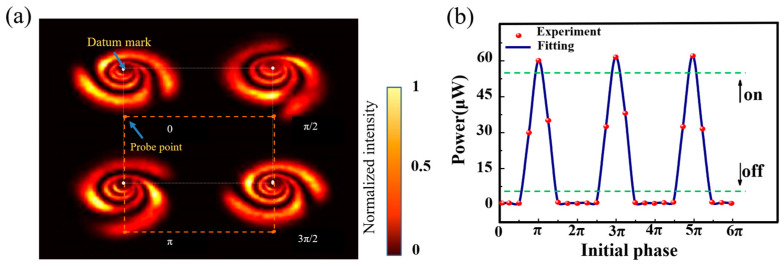
Operation principles of phase-triggered optical switching: (**a**) diffraction rings at different initial phases (Orange dotted lines: indicating the relative position of the probe point and datum mark); (**b**) power variation at the probe point with an increasing initial phase (Green dotted lines: threshold of “on” and “off” level signal).

It was verified that the interference beam can be optically switched in the radial direction. Then, we verified the optical switcher in the angular direction. Figure 10a shows the intensity distribution at fixed power when the interference beam has different initial phases. When an aperture was placed at the orange dots position of Figure 10a, the initial phase of the interference beam was continuously modulated through the phase interval of π/4, and the power received by the aperture was recorded. The data curve is shown in Figure 10b. The purpose of the optical switcher in the angular direction was achieved by regulating the initial phase. The optical switcher in the angular direction had a more uniform peak and valley, regardless of the number of diffraction rings.

**Figure 11:**
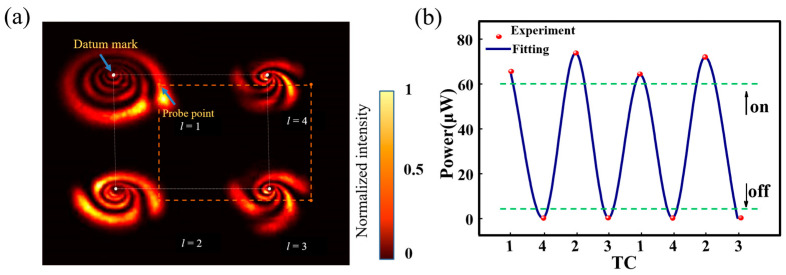
Operation principles of TC-triggered optical switching: (**a**) diffraction rings at different TC (Orange dotted lines: indicating the relative position of the probe point and datum mark); (**b**) power variation at probe point with changing TC (Green dotted lines: threshold of “on” and “off” level signal).

When the incident light power is the same, as the topological charge increases, the number of diffraction rings will decrease. This is because the nonlinear phase shift required to add a diffraction ring is 2π, so the amount of decrease is the absolute value of the topological charge change. Each increase in the topological charge number increases the phase required for the vortex optical phase rotation by 2π, and eventually both cancel each other out. As shown in Figure 11a, when the incident power was 25 mW, changing the topological charge caused changes in the diffraction pattern. When the diaphragm was placed at the orange dots position, as the topological load changed clockwise from 1-4-2-3-1, the diaphragm position underwent continuous change in peak and valley. The data results are shown in Figure 11b. When the tail was at the diaphragm position, if a threshold value was set as the critical point of the switch, the purpose of the optical switch was achieved by modulating the topological load of the incoming light.

### 4.3. Optical Logic Gate Based on Power-Phase Modulation

Using power and phase optical switches, we designed a set of optical logic gates that encoded the light intensity and the initial phase into data signals 1 and 2, respectively, and realized the optical logic AND gate, OR gate, and NOT gate. As show in Table 1, we defined the logic signal representation of the AND gate, OR gate, and NOT gate. For the AND gate, the power of incident light, as signal 1, 16 mW was defined as logic 1, while 7 mW was logic 0. The initial phase as signal 2, 3π/2 was defined as logic 0, while 0 was logic 1. For the OR gate, the power of incident light, as signal 1, 16 mW was defined as logic 1, while 7 mW was logic 0. The initial phase as signal 2, 3π/2 was defined as logic 0, while 0 was logic 1. For the NOT gate, the phase of incident light, as signal 2, 0 was defined as logic 1, while 3π/2 was logic 0.

**Figure 12:**
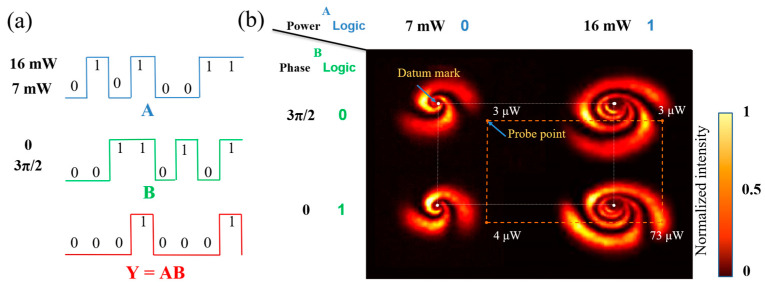
Logic AND gate based on modulated power-phase: (**a**) the waveform of logic AND gate (input: A and B; output: Y) and expression; (**b**) experimental result of power-phase modulated AND gate (Orange dotted lines: indicating the relative position of the probe point and datum mark).

As shown in Figure 12a, the operation rule of the AND gate is Y=AB, so both signal 1 and signal 2 are 1 and the output is 1. As shown in Figure 12b, we placed the diaphragm on the orange dots, and when the inputs were (7 mW, 3π/2), (7 mW, 0), and (16 mW, 3π/2), the values of the orange dots measured by a power meter were 3 μW, 4 μW, and 3 μW, respectively, which were lower than the threshold value of 50 μW, so the output was logic 0. When the input was (16 mW, 0), the value of the orange dot measured by a power meter was 73 μW, exceeding the threshold value of 50 μW, so the output was logic 1.

**Figure 13:**
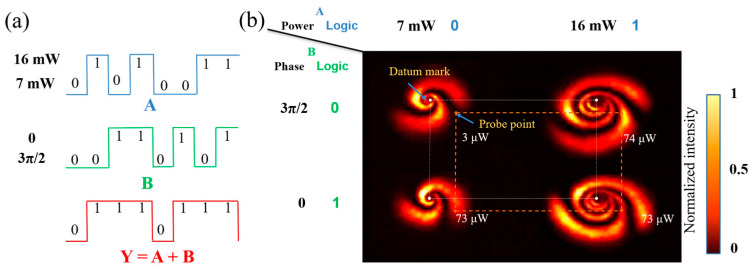
Logic OR gate based on modulated power-phase: (**a**) the waveform of logic OR gate (input: A and B; output: Y) and expression; (**b**) experimental result of power-phase modulated OR gate (Orange dotted lines: indicating the relative position of the probe point and datum mark).

As shown in Figure 13a, the operation rule of the OR gate is Y=A+B, so both signal 1 and signal 2 are 1 and the output is 1. As shown in Figure 13b, we placed the diaphragm on the orange dots, and when the input was (7 mW, 3π/2), the value of the orange dot measured by a power meter was 3 μW, lower than the threshold value of 50 μW, so the output was logic 0. When the inputs were (7 mW, 0), (16 mW, 3π/2), and (16 mW, 0), the values of the orange dots measured by a power meter were 73 μW, 74 μW, and 73 μW, respectively, exceeding the threshold value of 50 μW, so the output was logic 1.

**Figure 14:**
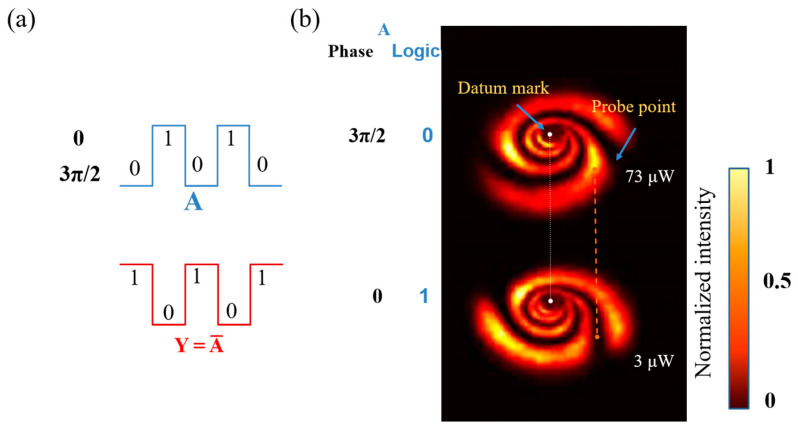
Logic NOT gate based on modulated power-phase: (**a**) the waveform of logic NOT gate (input: A; output: Y) and expression. (**b**) experimental result of power-phase modulated NOT gate (Orange dotted lines: indicating the relative position of the probe point and datum mark).

As shown in Figure 14a, the operation rule of the NOT gate is $Y=\bar{A}$, so signal 1 is 0 and the output is 1. As shown in Figure 14b, we placed the diaphragm on the orange dots, and when A = 3π/2 with the fixed power, the value of the orange dot measured by a power meter was 73 μW, exceeding the threshold value of 50 μW, so the output was logic 1. When A = 3π/2 with the fixed power, the value of the orange dot measured by a power meter as 3 μW, which was lower than the threshold value of 50 μW, so the output was logic 0.

Therefore, based on the experimental results and the logic definition of Table 1, we obtained the truth table about the designed AND gate, OR gate, and NOT gate, as shown in Table 2.

Overviewing the above Figure 12, Figure 13 and Figure 14 and Table 2, the results show that the basic logic AND gate, OR gate, and NOT gate can be realized by jointly adjusting the radial light intensity and the angular initial phase. These logic gates verified the practical application of the nonlinear diffraction method.

### 4.4. Optical Logic Gate Based on TC-Phase Modulation

We designed a set of optical logic gates based on the TC and the phase, encoding the TC and initial phase into data signals 1 and 2, respectively, and implementing optical logic AND gates, OR gates, and NOT gates. As shown in Table 3, we defined the logical signal representation of the AND gate, the OR gate, and the NOT gate. For the AND gate and the OR gate, TC *l* = 2, which was the power of the incident light as signal 1, defined as logic 0, and *l* = 1 is logic 1. For signal 2, the initial phase of 0 was defined as logic 0, and π was logic 1. For the NOT gate, the initial phase was signal 2, 0 was defined as logic 0, and π was logic 1.

**Figure 15:**
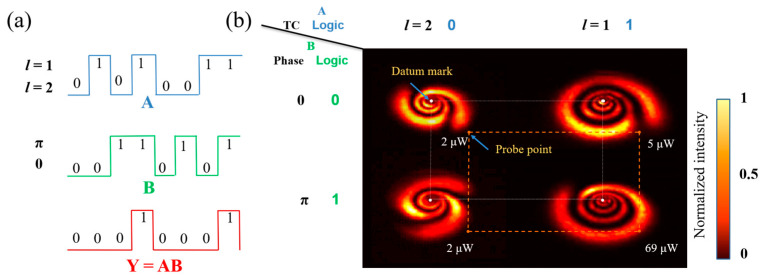
Logical AND gate based on modulated TC-phase: (**a**) the waveform of logic AND gate (input: A and B; output: Y) and expression; (**b**) experimental result of TC-phase modulated AND gate (Orange dotted lines: indicating the relative position of the probe point and datum mark).

As shown in Figure 15a, the operation rule of the AND gate is Y=AB, so both signal 1 and signal 2 are 1 and the output is 1. As shown in Figure 15b, we placed the diaphragm on the orange dots, and when the inputs were (*l* = 2, 0); (*l* = 2, π); (*l* = 1, 0), the values of the orange dots measured by a power meter were 2 μW, 2 μW, and 5 μW, respectively, which were lower than the threshold value of 50 μW, so the output was logic 0. When the input was (*l* = 1, π), the value of the orange dot measured by a power meter was 69 μW, exceeding the threshold value of 50 μW, so the output was logic 1.

**Figure 16:**
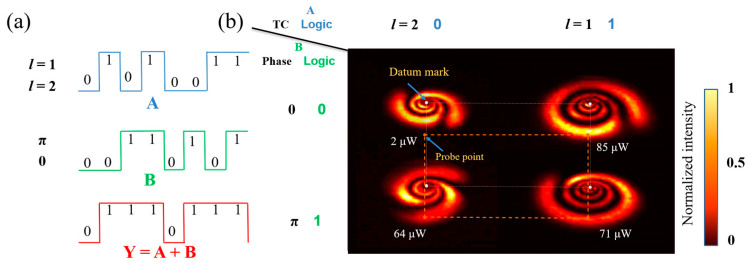
Logical OR gate based on modulated TC-phase: (**a**) the waveform of logic OR gate (input: A and B; output: Y) and expression; (**b**) experimental result of TC-phase modulated OR gate (Orange dotted lines: indicating the relative position of the probe point and datum mark).

As shown in Figure 16a, the operation rule of the OR gate is Y=A+B, so both signal 1 and signal 2 are 1 and the output is 1. As shown in Figure 16b, we placed the diaphragm on the orange dots, and when the input was (*l* = 2, 0), the value of the orange dot measured by a power meter was 2 μW, which was lower than the threshold value of 50 μW, so the output was logic 0. When the inputs were (*l* = 2, π), (*l* = 1, 0), (*l* = 1, π), the values of the orange dots measured by a power meter were 64 μW, 85 μW, and 71 μW, respectively, exceeding the threshold value of 50 μW, so the output was logic 1.

**Figure 17:**
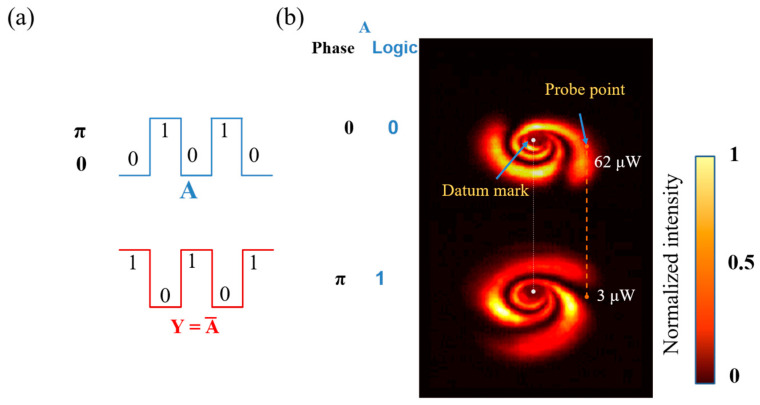
Logical NOT gate based on modulated TC-phase: (**a**) the waveform of logic NOT gate (input: A; output: Y) and expression; (**b**) experimental result of TC-phase modulated NOT gate (Orange dotted lines: indicating the relative position of the probe point and datum mark).

As shown in Figure 17a, the operation rule of the NOT gate is $Y=\bar{A}$, so signal 1 is 0 and the output is 1. Figure 17b shows that we placed the diaphragm on the orange dots and when A = 0 with the fixed power, the value of the orange dot measured by a power meter was 62 μW, exceeding the threshold value of 50 μW, so the output was logic 1. When A = π with the fixed power, the value of the orange dot measured by a power meter was 3 μW, which was lower than the threshold value of 50 μW, so the output was logic 0.

Therefore, based on the experimental results and the logical definition of the Table 3, we obtained the truth table about the designed AND gate, OR gate, and NOT gate, as shown in Table 4.

Overviewing the above Figure 15, Figure 16 and Figure 17 and Table 4, the results show that the basic logic AND gate, OR gate, and NOT gate can be realized by jointly adjusting the TC and the angular initial phase. These logic gates verify the practical application of the nonlinear diffraction method.

## 5. Discussion

Due to the SSPM effect of the MoS_2_ dispersion related to the incident light intensity, when the Gaussian vortex superposed interference light is used as the incident light, the MoS_2_ dispersion can be excited to produce the diffraction pattern, and the size and position of the diffraction pattern can be regulated by changing the light intensity and the initial phase. We encoded the light intensity and the initial phase, respectively, into signal 1 and signal 2, modulated the signal by dynamically changing the output power of the laser and the spiral phase diagram of SLM, and finally realized the optical logic AND gate, OR gate, and NOT gate through joint modulation. In comparison with other optical logic gates based on nonlinear phase modulation, which only achieve single-dimensional power modulation, by utilizing the non-uniform intensity distribution of a Gaussian vortex interference beam to stimulate the nonlinear diffraction of 2D material in a communication waveband, we realized a series of spatial optical logic gates with multiple input parametric form, such as the intensity-phase and the TC-phase. The extra degrees of freedom provided more flexibility and maneuverability of all-optical logic gates. All-optical logic gates based on SSPM might develop with new nonlinear materials, degrees of modulation, and coding schemes. Among the large family of 2D materials, many of them, such as graphene, graphdiyne oxide, and MoSe2, possess excellent optical nonlinear response and are suitable for all-optical logic gates. Because the SSPM diffraction pattern strongly depend on the incident beam’s intensity distribution, the other spatial structure beams with a non-uniform wavefront, such as Hermite–Gaussian beams, cylinder vector beams, Bessel beams, and Airy beams, also have great potential to serve as the carrier beam in all-optical logic gates. Moreover, this may provide new degrees of modulated freedom for logic coding. These novel optical logic gates are waiting to be explored and have great potential in all-optical networks and all-optical information processing.

## 6. Conclusions

In this study, we proposed and designed two types of novel optical logic gates based on the SSPM effect. When we used the Gauss vortex superposed interference light to excite the SSPM effect of MoS_2_ dispersion, the size and position of the nonlinear diffraction rings were related to the incident intensity, the initial phase, and TC of vortex beam. Therefore, the diffraction intensity distribution through the MoS_2_ nanosheets dispersions not only had diffraction rings in the radial direction, but also tails in the angular direction. Using this characteristic, we realized two groups of optical logic gates based on power-phase and TC-phase joint modulation for the first time, including the logic AND gate, OR gate, and NOT gate. In this study, the SSPM effect generated by the interference beam was initially simulated at. Then, the corresponding experiments were carried out and the results were compared with the simulation results; the optical pattern was collected and a truth table analysis of the optical logic gate was carried out. The results showed that Gaussian vortex superposition interference light can realize optical logic gates. This has great application potential in optical logic operation and all-optical signal processing.

## Figures and Tables

**Table 1:** Logic gates input signal.

	AND		OR		NOT	
	Logic 0	Logic 1	Logic 0	Logic 1	Logic 0	Logic 1
Signal 1: power(I)	7 mW	16 mW	7 mW	16 mW	--	--
Signal 2: phase(θ)	3π/2	0	3π/2	0	3π/2	0

**Table 2:** Three logic gates of the experimental results and the truth table.

AND								
	Experimental				Truth Table			
I	7 mW	16 mW	7 mW	16 mW	0	1	0	1
θ	3π/2	3π/2	0	0	0	0	1	1
output	3 μW	3 μW	4 μW	73 μW	0	0	0	1
**OR**								
	**Experimental**				**Truth Table**			
I	7 mW	16 mW	7 mW	7 mW	0	1	0	1
θ	3π/2	3π/2	0	0	0	0	1	1
output	3 μW	73 μW	74 μW	73 μW	0	1	1	1
**NOT**								
	**Experimental**				**Truth Table**			
θ	3π/2		0		0		1	
output	73 μW		3 μW		1		0	

**Table 3:** Logic gates input signals.

	AND		OR		NOT	
	Logic 0	Logic 1	Logic 0	Logic 1	Logic 0	Logic 1
Signal 1: TC	*l* = 2	*l* = 1	*l* = 2	*l* = 1	--	--
Signal 2: phase(θ)	0	π	0	π	0	π

**Table 4:** Three logic gates based on the experimental results and the truth table.

AND								
	Experimental				Truth Table			
TC	*l* = 2	*l* = 1	*l* = 2	*l* = 1	0	1	0	1
θ	0	0	π	π	0	0	1	1
output	2 μW	2 μW	5 μW	69 μW	0	0	0	1
**OR**								
	**Experimental**				**Truth Table**			
TC	*l* = 2	*l* = 1	*l* = 2	*l* = 1	0	1	0	1
θ	0	0	π	π	0	0	1	1
output	2 μW	64 μW	85 μW	71 μW	0	1	1	1
**NOT**								
	**Experimental**				**Truth** **Table**			
θ	0		π		0		1	
output	62 μW		3 μW		1		0	

## Data Availability

Data are contained within the article.
